# The relationship between the visual evoked potential and the gamma band investigated by blind and semi-blind methods

**DOI:** 10.1016/j.neuroimage.2011.03.008

**Published:** 2011-06-01

**Authors:** Camillo Porcaro, Dirk Ostwald, Avgis Hadjipapas, Gareth R. Barnes, Andrew P. Bagshaw

**Affiliations:** a Institute of Neuroscience, Newcastle University, Medical School, Framlington Place, Newcastle upon Tyne NE2 4HH, UK; b School of Psychology and Birmingham University Imaging Centre (BUIC), University of Birmingham, Birmingham, UK; c Laboratory of Electrophysiology for Translational NeuroScience (LET'S), ISTC-CNR, Fatebenefratelli Hospital – Isola Tiberina, Rome, Italy; d Department of Neurology and Bernstein Centre for Computational Neuroscience, Charite´, Berlin, Germany; e Donders Institute for Brain, Cognition and Behaviour, Radboud University, Nijmegen, The Netherlands; f Wellcome Trust Centre for Neuroimaging, UCL, London, WC1N 3BG, UK

**Keywords:** Visual Evoked Potential (VEP), Electroencephalography (EEG), Independent Component Analysis (ICA), Functional Source Separation (FSS), Induced Visual Gamma (IVG), Gamma Band Activity (GBA)

## Abstract

Gamma Band Activity (GBA) is increasingly studied for its relation with attention, change detection, maintenance of working memory and the processing of sensory stimuli. Activity around the gamma range has also been linked with early visual processing, although the relationship between this activity and the low frequency visual evoked potential (VEP) remains unclear. This study examined the ability of blind and semi-blind source separation techniques to extract sources specifically related to the VEP and GBA in order to shed light on the relationship between them. Blind (Independent Component Analysis—ICA) and semi-Blind (Functional Source Separation—FSS) methods were applied to dense array EEG data recorded during checkerboard stimulation. FSS was performed with both temporal and spectral constraints to identify specifically the generators of the main peak of the VEP (P100) and of the GBA. Source localisation and time-frequency analyses were then used to investigate the properties and co-dependencies between VEP/P100 and GBA. Analysis of the VEP extracted using the different methods demonstrated very similar morphology and localisation of the generators. Single trial time frequency analysis showed higher GBA when a larger amplitude VEP/P100 occurred. Further examination indicated that the evoked (phase-locked) component of the GBA was more related to the P100, whilst the induced component correlated with the VEP as a whole. The results suggest that the VEP and GBA may be generated by the same neuronal populations, and implicate this relationship as a potential mediator of the correlation between the VEP and the Blood Oxygenation Level Dependent (BOLD) effect measured with fMRI.

## Research Highlights

► ICA and FSS are able to extract sources specifically related to the VEP/P100 and GBA. ► Localisation and frequency analyses show co-dependencies between VEP/P100 and GBA. ► Trial by trial induced GBA covaries with VEP amplitude. ► VEP and GBA may be generated by the same neuronal populations. ► VEP and GBA relationship may underlie the relationship between VEP and BOLD.

## Introduction

Recording electric or magnetic fields from the scalp using electro- or magneto-encephalography (EEG/MEG) is one of the primary ways in which human brain activity can be characterised. However, the signals measured at the scalp are a mixture of the contributions from multiple generators or sources, added to background activity and system noise, meaning that it is often difficult to identify and study the dynamic activity of generators of interest at the level of the electrode/sensor space. Although the most common method of overcoming this limitation is time-domain averaging with or without source localisation (Key et al., 2005, Michel et al., 2004), source separation algorithms are becoming increasingly widely accepted as a way of extracting and investigating the different neuronal sources that contribute to the measured scalp signal. The advantage of blind or semi-blind source separation techniques compared to more restricted analyses such as time-domain averaging lies in their ability to explore the data and extract dependencies that were not expected a priori. They also provide a means of investigating non-phase locked oscillatory processes and improving the signal-to-noise ratio (SNR) of the sources of interest by allowing the extraction of a specific part of the signal.

Blind Source Separation (BSS) algorithms make some assumptions about the statistical properties of the sources contributing to the measured signal, but within the constraints of those assumptions are able to unmix or decompose the scalp signal into a number of underlying sources. The most common source separation technique in terms of EEG and MEG is Independent Component Analysis (ICA, Comon, 1994; Hyvärinen, 1999, Hyvärinen et al., 2001), which contains no prior information regarding the signals. Recently, a new semi Blind Source Separation (s-BSS) method has been developed and has shown some promising results. Functional Source Separation (FSS, Barbati et al., 2006; Tecchio et al., 2007) is an extension of ICA that incorporates prior information into the decomposition, resulting in a single component which maximises a temporal or spectral constraint of interest.

The advantages of source separation techniques are most evident when dealing with trial-by-trial variations of electrophysiological signals (Makeig et al., 2002, 2004a,b; Porcaro et al., 2010), or with other low amplitude and noisy aspects of the scalp signals such as oscillations in the gamma band (~ 30–90 Hz, Hadjipapas et al., 2007; Barbati et al., 2008, Muthukumaraswamy et al., 2010). Gamma Band Activity (GBA) is increasingly widely studied because of its links with processes as disparate as the binding of cortical regions and stimulus features, attention, change detection, maintenance of working memory and the processing of sensory stimuli (Gray et al., 1989; Fries et al., 2001; Pesaran et al., 2002; Womelsdorf et al., 2006; see Tallon-Baudry, 2003, Ward, 2003 and Fries et al., 2007 for reviews). Activity around the gamma range has also been linked with early visual processing (Tzelepi et al., 2000; Sannita et al., 2001; Hadjipapas et al., 2007; Perfetti et al., 2007), although the relationship between this activity and the low frequency Visual Evoked Potential (VEP) remains unclear, with most studies concentrating on one aspect or the other (although see Muthukumaraswamy et al., 2010). Given the prevalence of these different responses to visual stimuli, and the amount of work that has been done to characterise their properties, it is perhaps surprising that more is not known about how the two are related. For example, are they generated by the same neuronal pools, or do they represent the stimulus responses of independent but spatially co-localised populations? Is there a link between the VEP/P100 and the GBA?

These questions have a wider significance when considering the combination of EEG with functional magnetic resonance imaging (fMRI). Although a number of lines of evidence from animal and human studies suggest that the tightest coupling between EEG and BOLD occurs in the gamma band (see, for example, Logothetis et al., 2001, Niessing et al., 2005, Muthukumaraswamy and Singh, 2008, Zaehle et al., 2009), most combined EEG-fMRI studies in humans are interested in and restricted to the lower frequencies of evoked potentials (see Debener et al., 2006 for a review). In general, this has also proved fruitful, although even when restricting the analysis to low frequencies the goal of investigating co-dependencies between EEG and fMRI is complicated since many potential features can be investigated (Ostwald et al., 2010). For example, Porcaro et al. (2010) observed a significant correlation between the mean area of the VEP and that of the BOLD response, but not when considering their respective amplitudes. What extra neurophysiological significance was carried by the area of the VEP was not clear. However, it is evident that if EEG-fMRI is to achieve its potential as a neuroimaging methodology, the nature of the link between the two signals must be more easily interpretable at all frequencies.

In order to address these issues, and to investigate the ability of different methodologies to extract the sources of interest, blind (ICA) and semi-Blind (FSS) source separation methods were applied to dense-array EEG data recorded during checkerboard stimulation. FSS was performed with both temporal (Porcaro et al., 2008, 2009, 2010) and spectral (Porcaro et al., 2008; Barbati et al., 2008) constraints to identify specifically the generators of the main peak of the VEP/P100 and of the GBA. Source localisation and time-frequency analyses, single trial and on average data, were then used to investigate the properties and co-dependencies of the sources identified with the different methods to build up a picture of the link between VEP/P100 and GBA.

## Materials and methods

### Participants and data acquisition

Four subjects (1 female, age range 26–33) were paid for their participation. Written informed consent was obtained and the protocol was approved by the Research Ethics Board of the University of Birmingham.

A full field, high (100%) contrast, low spatial frequency (~ 0.5 cycles per degree) checkerboard stimulus was presented with a reversal rate of 2 Hz (i.e. the checkerboard was presented for 500 ms before reversing). This spatial frequency was chosen to be similar to the standards for generating VEPs used clinically (see for example Odom et al., 2004), and may not be optimal for the generation of GBA. However, the primary concern for the current study is that the stimulus is capable of generating both VEPs and GBA, thereby allowing the relationship between the two to be investigated. Stimuli were displayed on a computer monitor with a refresh rate of 80 Hz using Presentation (Neurobehavioral Systems Inc., CA, USA), approximately 80 cm from the seated subject, in blocks of 5 s with a 10 s gap between successive blocks. Stimuli were synchronised with the screen refresh. Participants were instructed to fixate on a central red dot which was present between checkerboard stimuli and to minimise blinking whilst the checkerboard was presented. Each run consisted of twenty blocks, and took approximately 5 min. In total, 600 VEPs were collected (10 VEPS per block, 20 blocks per run, 3 runs). EEG data were acquired using a 128 channel BioSemi Active Two EEG system (BioSemi, Amsterdam, the Netherlands), with electrodes placed in a nylon cap according to the 10–5 system (Oostenveld and Praamstra, 2001). The data were sampled at 2048 Hz with a linked mastoid reference.

The data from the runs were concatenated, re-referenced to common average and down-sampled to 512 Hz. The data were also low pass filtered (100 Hz) prior to the off line analysis. Some trials were discarded by visual inspection in each subject due to the movement artefacts, the majority of these trials were discarded at the beginning and the end of each runs. After this procedure we had 446, 548, 567 and 490 trials respectively for subjects 1, 2, 3 and 4. For the further analysis we used a consistent number of trials across the subjects using 400 trials for each subject. The analysis strategy aimed at extracting signals generated only in response to the stimulus.

### Source separation algorithms

Two methods of source separation were used: blind ICA (fastICA, Hyvärinen, 1999; Hyvärinen et al., 2001; Barbati et al., 2004) and semi-blind FSS (Barbati et al., 2006; Tecchio et al., 2007). FSS was performed with both temporal (Porcaro et al., 2008, 2009, 2010) and spectral (Barbati et al., 2008; Porcaro et al., 2008) constraints to extract the sources of interest.

#### Independent Component Analysis (ICA)

ICA (Comon, 1994) is a generative ‘latent variable’ model that describes how the observed data are generated by a process of mixing the underlying unknown sources; the sources (ICs) are assumed to be statistically independent and non-Gaussian. Since the observed mixed signals will tend to have more Gaussian amplitude distributions, ICA strives to find a separation matrix that minimizes the Gaussianity of the results, thus optimally separating the signals. For this purpose, we assumed the set of observed EEG signals to be generated by the mixing model:

(1) x(t)=As(t)

where t = 0,1,2,… is the discrete sampling time; **x**(*t*) = [*x*_1_(*t*), …, *x*_*m*_(*t*)] is the *m*-dimensional vector of the observed signal recorded by *m* electrodes; ***A*** is an *m* × *n* (with*n* ≤ *m*) unknown full-rank mixing matrix; **s**(*t*) = [*s*_1_(*t*), …, *s*_*n*_(*t*)]^*T*^ is the *n*-dimensional unknown vector of the sources. The model is approached by processing electrode signals by an ICA demixing system described in the form:

(2) $$IC\left(t\right)=Wx\left(t\right)$$

where *IC*(*t*) = [*IC*_1_(*t*), …, *IC*_*n*_(*t*)]^*T*^ is the *n*-dimensional vector of the estimated ICs and W is the separation matrix, i.e., the estimate of the inverse of the unknown mixing matrix ***A***, up to permutation and scaling:

(3) $$W={\hat{A}}^{-1}\text{.}$$

We used the FastICA algorithm proposed by Hyvärinen (Hyvärinen, 1999, Hyvärinen et al., 2001).

In the case of a large number of channels (128 in this case) a direct extraction of all the ICs would have been extremely time consuming and component selection extremely challenging. We pursued a dimensionality reduction by applying an optimized procedure to select a *k* ICs-subset such that the corresponding explained variance was at least 95% (Salustri et al., 2005). In our case on average 31 ICs [range 20–39] resulted in a mean explained variance ± standard deviation of 97.9% ± 1.4% (Table 1).

##### Selection of sources

From the 20–39 ICs, Event Related Spectral Perturbation (ERSP) triggered by rest (from − 5 to 0 s) and task (from 0 to + 5 s) was used to identify ICs of interest. Specifically, ICs were identified which had an increased power in the gamma band. In three subjects only one IC was identified (for subjects 1, 2 and 4 ICs 20, 12 and 14 were selected), whilst three ICs were selected for subject 3 (ICs 6, 8 and 14). After the identification of ICs, the data at the scalp electrodes were obtained by retroprojecting the selected ICs:

(4) $$EEG_{IC_{k}}=A_{k}IC_{k}$$

where **A**_*k*_ is the estimated mixing vector (matrix **A** of Eq. (1)) for the source **IC**_*k*_ and **EEG**_*IC*_*k*__ is the resulting **IC**_*k*_ retro-projection on the channels space.

#### Functional Source Separation (FSS)

As in the ICA approach, FSS starts from an additive hidden source model of the type in Eq. (1), where **X** represents the observed EEG data, **S** are the underlying unknown sources and **A** is the source-sensor coupling matrix to be estimated. Additional information to a standard ICA model is used to bias the decomposition algorithm towards solutions that satisfy physiological assumptions. In other words, the aim of FSS is to enhance the separation of relevant signals by exploiting some a priori knowledge without renouncing the advantages of using only information contained in the original signal waveforms. A modified (with respect to standard ICA) contrast function is defined:

(5) $$F=J+\lambda R_{\mathrm{FS}}$$

where ***J*** is the statistical index normally used in ICA, whilst *R*_*FS*_ accounts for the prior information used to extract a single source. According to the weighting parameter λ it is possible to adjust the relative weight of these two aspects. In this study, λ was chosen to be equal to 1000 in all cases, as detailed in Porcaro et al. (2008). Briefly, *λ* was chosen to both minimise computational time and maximise *R*_*FS*_. Moreover, since prior information about the sources may also be described by a non-differentiable function, the new contrast function ***F*** is optimised by means of simulated annealing (Kirkpatrick et al., 1983; Barbati et al., 2006 Appendix A). This does not require the use of derivatives, and performs global optimisation, whilst the gradient-based algorithms usually employed in ICA only guarantee local optimisation. This scheme gives us the possibility to extract only one component that maximises the functional behaviour in agreement with the functional constraint. In the present work, consistent with the VEP and GBA under investigation, two ad-hoc functional constraints were maximised. The first constraint was related to the principal peak (P100) of the VEP. The second was related to the GBA and a maximisation of the difference in Power (Power Spectral Density, PSD) between rest and task was used. These constraints are discussed in more detail below.

##### Temporal functional constraint

The functional constraint *R* was defined as:

(6) $$R(FS_{P100})=\sum_{t_{k}-\Delta_{1}t_{k}}^{t_{k}+\Delta_{2}t_{k}}\left|EA(FS_{P100},t)\right|-\sum_{-100}^{0}\left|EA(FS_{P100},t)\right|$$

with the evoked activity, EA, computed by averaging signal epochs of the source FS_P100_, triggered on the visual stimulation (t = 0); *t*_*k*_ is the time point with the maximum electric potential around 100 ms after the stimulus onset on the maximal original EEG channel; *Δ*_1_*t*_*k*_(*Δ*_2_*t*_*k*_) is the time point corresponding to a signal amplitude of 50% of the maximal value before (after) *t*_*k*_. The baseline was computed in the time interval from − 100 to 0 ms. The precise value of each latency *t*_*k*_ was chosen for each subject, corresponding to the maximum electric potential in the time interval of interest (80–120 ms), see also Porcaro et al. (2010). The source was then retro-projected to obtain its electric potential distribution at the scalp electrodes:

(7) $$EEG_{\mathrm{FS}P100}=A_{P100}FS_{P100}$$

where $A_{P100}$ is the estimated mixing vector (matrix **A** of Eq. (1)) for the functional source and $EEG_{\mathrm{FS}P100}$ are the retro-projections on the electrodes of the estimated FS_P100_.

##### Spectral functional constraint

To investigate the GBA, the following ad-hoc functional constraint R was used:

(8) $$R(FS_{\gamma })=\frac{\sum_{\gamma }PSD(FS_{\gamma }){\;}_{\text{Stimulus}}-\sum_{\gamma }PSD(FS_{\gamma }){\;}_{\text{No-Stimulus}}}{\sum_{\gamma }PSD(FS_{\gamma }){\;}_{\text{No-Stimulus}}}\text{.}$$

This constraint computes the difference in the Power Spectrum Density (PSD) between Stimulus (from 0 to 5 s of each trial, t = 0 corresponding to the stimulus onset) and No-Stimulus (from − 5 to 0 s of each trial) periods in the γ frequency band (30–90 Hz). This difference is then normalised with respect to the GBA in the No-Stimulus period (Barbati et al., 2008). The source was then retro-projected to obtain its electric potential distribution at the scalp electrodes:

(9) $$EEG_{FS_{\gamma }}=A_{\gamma }FS_{\gamma }$$

where $A_{\gamma }$ is the estimated mixing vector (matrix **A** of Eq. (1)) for the functional source and $EEG_{\mathrm{FS\gamma}}$ are the retro-projections on the electrodes of the estimated ***FS***_γ_ .

### Data evaluation

To evaluate the quality of the data following ICA and FSS, four criteria were used: the VEP, Source Localisation, Discrepancy and Time Frequency Dynamics applied on both averaged trials and single trial levels. In order to facilitate comparison, the analysed data were taken from a single electrode selected for each subject based on the maximum of the voltage field (Eq. (4) for ICA and Eqs. (7) and (9) for FSS with the time and spectral constraints respectively), at the latency of the P100 peak. The electrode nomenclature is according to the 10–5 electrode system (Oostenveld and Praamstra, 2001). The selected electrodes for each subject and for each method are shown in Table 2.

#### Visual Evoked Potential

After identification of the ICs with increased gamma activity during the task with respect to the rest period, extraction of FS_P100_ and FS_γ_, scalp topographies were obtained by retroprojection of the identified sources using Eq. (4) for ICA and Eqs. (7) and (9) respectively for the FSS with temporal and spectral constraint. In each subject the signals from the selected electrode were averaged based on the stimulus trigger to obtain the VEP. The grand average across subjects was also calculated.

#### Source localisation

Source localisation was performed using an equivalent current dipole (ECD) model, with a forward model consisting of four concentric conductive spheres (routine DIPFIT2 (Oostenveld and Oostendorp, 2002) of EEGLAB v6.01b, available at http://www.sccn.ucsd.edu/eeglab (Delorme and Makeig, 2004)). EEGLAB expresses ECD position in Talairach coordinates and projects them onto the Montreal Neurological Institute (MNI) template brain (Table 3). In order to test whether the localisation of the ECDs was comparable between methods, a repeated measures one-way ANOVA with levels $EEG_{IC_{k}}$, $EEG_{\mathrm{FS}P100}$, $EEG_{\mathrm{FS\gamma}}$ for X, Y and Z coordinates was performed.

#### Discrepancy

To determine whether the data after source selection contained any residual signal of interest, the discrepancy was defined as the difference between the original EEG data and the data obtained from Eq. (4) for ICA and Eqs. (7) and (9) for FSS (respectively for the time constraint and the spectral constraint):

(10) $$\begin{array}{l} Discrepancy_{IC_{k}}=EEG-EEG_{IC_{k}}\\ Discrepancy_{FS_{P100}}=EEG-EEG_{FS_{P100}}\\ Discrepancy_{\mathrm{FS\gamma}}=EEG-EEG_{\mathrm{FS\gamma}}\text{.} \end{array}$$

A mean discrepancy index (DI) was calculated as the ratio between the mean R computed on the discrepancy matrix and the mean R across the EEG electrodes:

(11) $$DI=\frac{{\sum_{i}\bar{R}\left(Discrepancy_{\mathrm{Method}}\right)}^{2}}{{\sum_{i}\left(\bar{R}(EEG)\right)}^{2}}\text{.}$$

$\bar{R}$ is the reactivity index defined as:

(12) $$\bar{R}=\frac{R_{}}{\Delta_{2}t_{k}+\Delta_{1}t_{k}+1}\text{.}$$

*R* is defined as in Eqs. (6) and (8) and *t*_*k*_ refers to the latency of each extracted FS or ICs (see Table 2). The index *i* runs upon all the channels. To test how well the three methods were able to extract the VEP in the time domain, we examined the discrepancy associated with each method. A one-way repeated measure ANOVA of the absolute post-stimulus discrepancy time-series was calculated with data from the three methods.

#### VEP/P100 regression

One problem when looking at time frequency plots of stimulus induced changes is that such maps contain both the evoked and induced responses. In order to specifically examine the induced changes we took the average evoked response over all trials and regressed this out of each individual trial. This gives an estimate of the purely induced component of the response, within the limitations of the standard model of evoked potential generation which assumes that the EP and background activity are additive. Although still a matter of debate, with evidence for an alternative model whereby evoked responses are the result of phase resetting of ongoing activity (Min et al., 2007; Hanslmayr et al., 2007; Sayers et al., 1974; Basar et al., 1998; Schürmann and Başar, 2001), this evoked model has been demonstrated to be appropriate for early (< 175 ms) components of the VEP (Becker et al., 2008; Mazaheri and Jensen, 2006). The results obtained using this approach were labelled as ‘regressed’ in the figures.

#### Time frequency dynamics

The data subjected to the time frequency analysis were taken from the same electrodes as used to investigate the VEP (Table 2). Time-frequency analysis of the data was performed using a short-time Fourier analysis using Fast Fourier Transforms (FFTs) with a moving windows size of 256 samples (500 ms) wide as implemented in EEGLAB (Delorme and Makeig, 2004). Event-related spectral perturbation (ERSP—event-related spectral perturbation, a 2-D (frequency-by-latency) image of mean change in spectral power (in dB) from baseline (Makeig et al., 2004a, see Concept and Terms)) was computed for each electrode, and the results were compared amongst the methods. The time-frequency plot was thresholded at a bootstrap significance level of p = 0.01. To investigate whether the GBA was phase locked to stimulus presentation (i.e. evoked or induced) the ERSP was also calculated after trial averaging. This identified the evoked component, which was then deleted by the procedure described above from the single trial power in the original signal to leave an estimate of the induced GBA power. Moreover, to specifically examine the effect of the three methods ($EEG_{IC_{k}}$,$EEEG_{\mathrm{FS}P100}$ and $EEG_{\mathrm{FS\gamma}}$) on different frequency bands, ERSP was averaged for each trial within a frequency range of 8–13 Hz (Alpha Band), 14–30 Hz (Beta Band), 31–60 Hz (Low Gamma Band) and 61–90 Hz (High Gamma Band).

ERP image plots were used to visualise the trial-by-trial variability on the different power bands. Instead of simply summing all the data trials, trials are first sorted based in order of a relevant data or external variable, and then plotted as a colour-coded two dimensional image. Essentially, 2-D ERP images generalise 1-D ERP averaging (Makeig et al., 2004a, see Concept and Terms).

#### VEP/P100 vs. frequency bands

In order to investigate whether there was a relationship between the evoked response and the different frequency bands present in the EEG, a single trial (ST) comparison between the VEP/P100 and the different frequency bands was performed for the different methods ($EEG_{IC_{k}}$,$EEG_{\mathrm{FS}P100}$ and $EEG_{\mathrm{FS\gamma}}$). The ST VEP was ordered by the area under the VEP curve (the VEP was rectified (i.e. abs function in Matlab) and then averaged in the range of the VEP (i.e., between ~ 65 ms and ~ 145 ms according to the single subject variability)). In addition, the ST P100 peak was ordered by the area under the P100 peak (between ~ 80 ms and ~ 120 ms). Both areas were then normalised with respect to the size of the windows used in each subject. As well as these measures of the VEP, the ERSP for each trial was calculated and averaged within frequency ranges of 8–13 Hz (Alpha Band), 14–30 Hz (Beta Band), 31–60 Hz (Low Gamma Band) and 61–90 Hz (High Gamma Band) with baseline correction using the interval − 100 ms to 0 ms. The trial ordering based on P100 and VEP area was then also applied to the ERSP data in order to investigate, for example, whether trials with a large VEP/P100 also had a large ERSP in each of the specific frequency bands. In order to investigate the relationship between the induced activity and the VEP/P100 an optimally weighted version of the average evoked response was then removed from each trial of the ordered data described above. The time frequency analysis was therefore performed for evoked, induced and induced plus evoked data.

### Statistical analysis

All statistical analyses were carried out using SPSS 16.0 (SPSS Inc, Chicago IL, USA).

## Results

### Selection of sources

From the ICA decomposition of each subject, components displaying significant post stimulus GBA were selected and retro-projected to the scalp space (from 1 to 3 components). FSS results in only one source for each constraint, avoiding the need for component selection.

### Evaluation of the extracted sources

#### Visual Evoked Potentials and source localisation

**Fig. 1:**
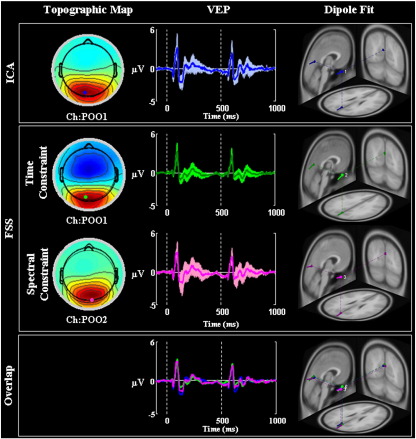
Topographic map, VEP and Dipole localisation across methods. For the grand average, the Topographic Map, VEP and Dipole localisation are shown for each method (**EEG**_*IC*_*k*__—first row, **EEG**_*FSP*100_—second row and **EEG**_*FSγ*_—third row). For the Dipole Fit, position and orientation of the ECD are shown superimposed on the MNI brain template in axial, coronal, and sagittal views. The last row shows the overlap across the methods for the VEP and dipole source localisation. The envelope indicates plus and minus one standard deviation around the VEP mean.

Firstly, the VEP (Fig. 1, second column) and its topographic map (Fig. 1, first column) and dipole source localisation (Fig. 1, third column) were examined. The topographic maps for all methods displayed a dipolar potential distribution (Fig. 1, first column) confirming the suitability of a single dipole model as an inverse solution strategy. Although the $EEG_{\mathrm{FS\gamma}}$ source was extracted from the data based solely on maximising the GBA, the Topographic Map, VEP and the dipole location derived from it were extremely consistent with those from the $EEG_{IC_{k}}$and $EEG_{\mathrm{FS}P100}$ sources (Fig. 1). A repeated measures one-way ANOVA with Greenhouse−Geisser correction for each ordinate individually revealed no significant localisation differences between methods (X-Ordinate: F(1.2,3.7) = 0.20, p = 0.72, Y-Ordinate: F(1.1,3.3) = 2.78, p = 0.19, Z-Ordinate: F(1.7,5.2) = 4.03, p = 0.09, see also Table 3).

#### Discrepancy

**Fig. 2:**
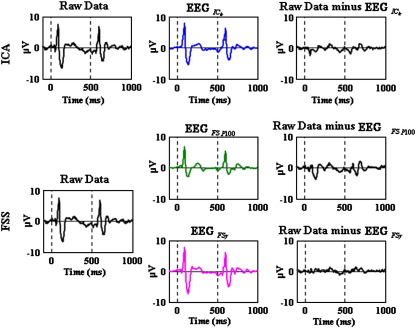
Discrepancy. For a representative subject and for **EEG**_*IC*_*k*__—first row, **EEG**_*FSP*100_—second row (top) and **EEG**_*FSγ*_—second row (bottom), averaged data are shown in the time window [− 100, 1000] ms (onset at t = 0 ms; reversal at t = 500 ms). Left: Raw Data. Centre: retro-projected data obtained by ICA (first row) or FSS (second row). Right: Raw Data minus **EEG**_*IC*_*k*__ (first row), Raw Data minus **EEG**_*FSP*100_ (second row—top) and Raw Data minus **EEG**_*FSγ*_ (second row—bottom).

Fig. 2 shows the results of the calculation of the discrepancy. It can be seen that all three methods were successful at extracting the signals of interest whilst leaving minimal residual activity (i.e. the extracted source described practically all of the evoked response contained in the original EEG data). As expected, since it is intended to extract only the P100, the $EEG_{IC_{k}}$did not extract all of the peaks of the VEP and there appear to be slight deviations at longer latencies (i.e. 140 ms). Interestingly, the discrepancy associated with $EEG_{\mathrm{FS\gamma}}$ appeared to be lower than for the other two techniques, suggesting that extracting a source that maximised the GBA was also able to account for the large majority of the signal at low frequency. All of the peaks of the VEP were accounted for in the $EEG_{\mathrm{FS\gamma}}$ method.

To confirm these observations, a one-way repeated measures ANOVA of the absolute post-stimulus time-series for levels $EEG_{IC_{k}}$ (mean 0.50, standard deviation 0.36), $EEG_{\mathrm{FS}P100}$ (mean 0.56, standard deviation 0.52) and $EEG_{\mathrm{FS\gamma}}$ (mean 0.39, standard deviation 0.32) with Greenhouse–Geisser correction for non-sphericity was performed. This revealed a significant omnibus effect (F(1.5, 765.3) = 61.8, p < 0.001). Further, all three pairwise comparisons revealed significant differences (p < 0.01) after Bonferroni correction for multiple comparisons. Together, these results indicate that the $EEG_{\mathrm{FS\gamma}}$method accounted for more of the stimulus-related activity than either the $EEG_{IC_{k}}$ or $EEG_{\mathrm{FS}P100}$ methods.

#### Time frequency dynamics

**Fig. 3:**
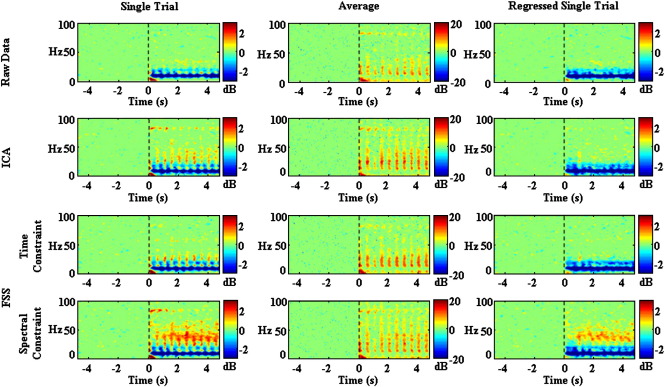
Induced gamma investigation by ERSP. Grand Average of ERSP for Raw Data—first row and for each method (**EEG**_*IC*_*k*__—second row, **EEG**_*FSP*100_—third row and **EEG**_*FSγ*_—fourth row). The left column shows the ERSP calculated for the single trials and then averaged. The middle column shows the ERSP for the averaged trials (i.e. ERSP of the average VEP). The right column shows the ERSP calculated for the regressed single trials and then averaged (note in this case the absence of the regular broad band transient which represents the VEP). The plots are thresholded at a bootstrap significance level of p = 0.01 such that effects below threshold are shown in green for the ERSP. The points after 0 (from 0 to 5 s) correspond to the time when a reversal checkerboard stimulus was presented on the screen (Stimulus—Task), and the points before 0 (from − 5 to 0 s) correspond to the time in which no reversal checkerboard stimulus was present (No-Stimulus—Rest).

For the ERSP, the gamma activity after the stimulus presentation was more evident in the blind and semi-blind methods than in the raw data (Fig. 3). As expected, since it is optimised to do so, the GBA was most robust in the $EEG_{\mathrm{FS\gamma}}$data. To investigate whether the GBA was phase locked to stimulus presentation (i.e. evoked or induced), the ERSP was also calculated after trial averaging (Fig. 3, first and second columns). Fig. 3 (third column) shows the estimate of the stimulus induced changes after the average evoked response has been regressed out (note the absence of the regular broad band transient). This procedure demonstrated the presence of both induced and evoked components to the GBA, which was most evident in the $EEG_{\mathrm{FS\gamma}}$data. It also shows very clearly the presence of a sustained, induced alpha band response to the stimulation block, which is evident with all processing methods. Such an alpha band desynchronisation is commonly observed with visual stimulation, and if the occipital alpha rhythm is considered an ‘idling’ state of visual cortex, it would correspond to a non-specific event-related activation of visual cortex (Pfurtscheller and Lopes da Silva 1999). From this point of view, it is conceptually different to the VEP and the GBA, which are explicit responses to stimulation.

**Fig. 4:**
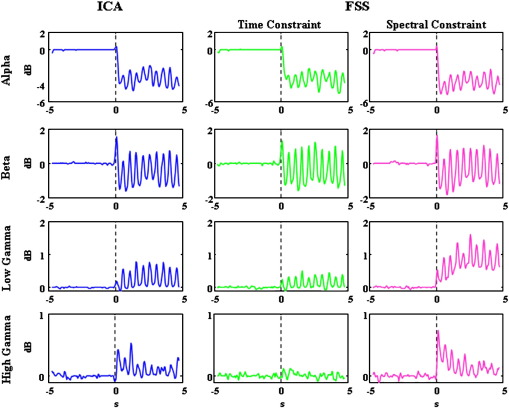
Alpha, Beta, Low Gamma and High Gamma activity. Grand Average across subjects of ERSP in the Alpha (8–13 Hz), Beta (14–30 Hz), Low Gamma (31–60 Hz) and High Gamma (61–90 Hz) bands as a function of time for all methods (**EEG**_*IC*_*k*_—_first column, **EEG**_*FSP*100_—second column and **EEG**_*FSγ*_—third column). The data in this figure refers to left column of Fig. 3.

Fig. 4 shows the average ERSP within the alpha (8–13 Hz), beta (14–30 Hz), low gamma (31–60 Hz) and high gamma (61–90 Hz) bands. As can be seen, characterisation of the different frequency bands was very consistent across methods, with the primary difference being the improved ability of $EEG_{\mathrm{FS\gamma}}$to extract GBA. In particular, a clear, sustained induced component of the low GBA was observed with $EEG_{\mathrm{FS\gamma}}$, but largely absent with the other methods.

Statistical analysis confirmed these observations. After filtering into the different frequency bands, the absolute post-stimulus ESRP underwent a two-way repeated measures ANOVA with factors ‘frequency band’ (alpha, beta, low gamma, and high gamma) and ‘method’ (Raw, $EEG_{IC_{k}}$, $EEG_{\mathrm{FS}P100}$, and $EEG_{\mathrm{FS\gamma}}$ Data) with Greenhouse–Geisser correction for non-sphericity. This revealed a significant main effect of ‘frequency band’ (F(1.4, 135.2) = 999.9, p < 0.001), a significant main effect of ‘method’ (F(2.6, 254.3) = 302.0, p < 0.001) and a significant interaction (F(4.6, 454.4) = 60.1, p < 0.001). Examining more specifically the effect of method, all pairwise comparisons other than the comparison of $EEG_{IC_{k}}$and $EEG_{\mathrm{FS}P100}$ (p = 0.71) revealed significant differences (p < 0.001) after Bonferroni correction for multiple comparisons based on the estimated marginal means.

As indicated by the significant interaction between the factors, the differences in absolute post-stimulus ERSP between the different processing methods were dependent on the frequency band investigated. Hence, we subsequently carried out a series of one-way ANOVAs comparing the processing methods for each frequency band individually. Of particular interest, in the low gamma band the effect of method was significant (F(1.5, 144.0) = 484.8, p < 0.001), indicating the improved GBA in the $EEG_{\mathrm{FS\gamma}}$ method.

#### VEP/P100 vs. frequency bands

**Fig. 5:**
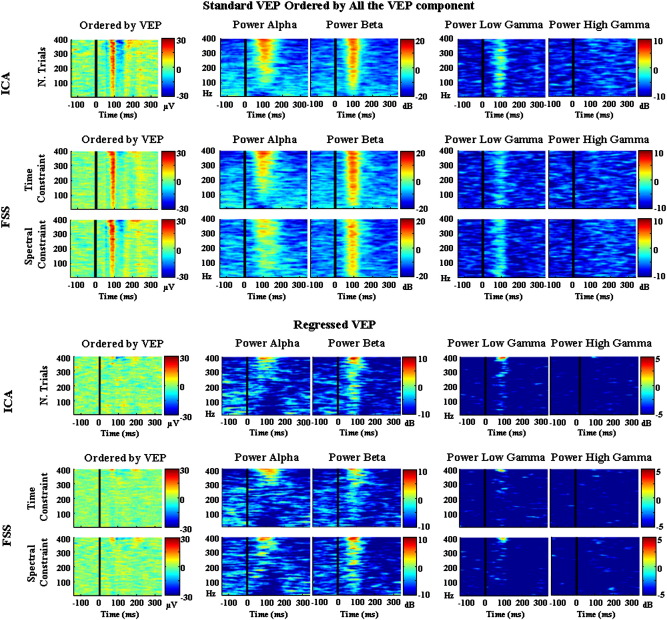
Single trial comparison—normalised VEP area. Grand Average across subjects – upper half – The single trial VEPs ordered by the area under the VEP curve (i.e. ~ 65 ms and ~ 145 ms). This ordering was then applied to the ERSP data for each of those trials within the frequency ranges of 8–13 Hz (Alpha Band), 14–30 Hz (Beta Band), 31–60 Hz (Low Gamma Band) and 60–90 Hz (High Gamma Band) amongst methods (**EEG**_*IC*_*k*__, **EEG**_*FSP*100_ and **EEG**_*FSγ*_). Lower half—as described above but after regressing out the evoked response.

**Fig. 6:**
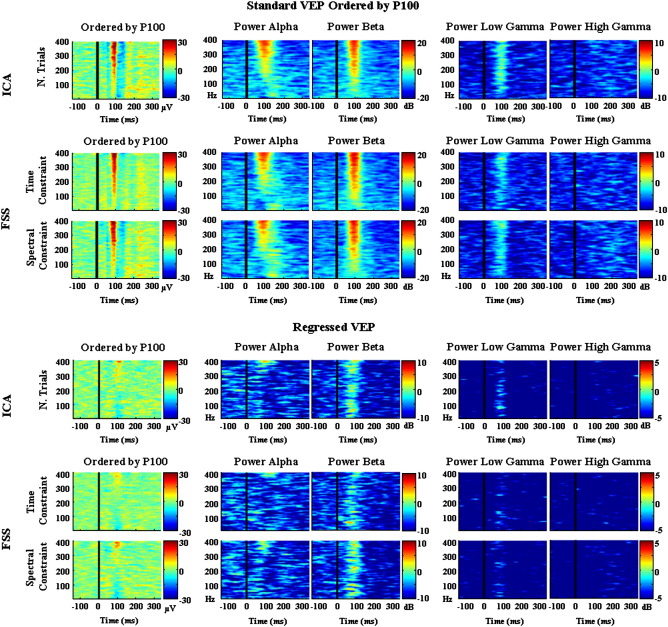
Single trial comparison—normalised P100 area. The same data used in Fig. 5 but ordered based on the area under the P100 peak (~ 80 ms and ~ 120 ms) and on the data after regressing out the evoked response (upper half and lower half respectively).

Figs. 5 and 6 show the ST comparisons between the normalised area of the VEP/P100 and the alpha, beta, low gamma and high gamma band ERSP for the different methods ($EEG_{IC_{k}}$,$EEG_{\mathrm{FS}P100}$, and $EEG_{\mathrm{FS\gamma}}$). The results for all of the methods are shown to confirm that the effects are consistent despite different data processing strategies.

The upper half of Fig. 5 (first column) shows the ERP image (Makeig et al., 2004a; 2004b, Delorme and Makeig, 2004) of the VEP single trials, with the trials ordered by the normalised area of the VEP. Columns 2 and 3 show the ERSP in the different frequency bands, with the trials ordered according to column one. From the figure it is clear that trials with a larger normalised VEP area show higher alpha and beta power, with a similar though less evident trend for the low gamma band. For the high gamma band this effect was not evident. The effect was observed consistently for all the methods.

Maintaining the same ST ordering model used above, the lower half of Fig. 5 (first column) shows the corresponding ERP image following removal of the average VEP through linear regression. This data therefore represents only the induced activity, whilst the data shown in the upper half of Fig. 5 contain both induced and evoked components. From the figure it is clear that the observations made for the upper half of Fig. 5 were also confirmed for the induced activity alone, indicating that the relationship between the VEP and GBA is not driven purely by frequency components of the evoked potential.

Fig. 6 used the same scheme as Fig. 5, except that the trials are ordered by the magnitude of the P100 peak alone, rather than the whole VEP. A similar link between P100 amplitude and spectral power can be seen as for the VEP, which is lost when the average VEP is regressed out (lower half of Fig. 6). This indicates that the link between P100 and spectral content is driven by the evoked component, with little or no contribution from the induced component.

**Fig. 7:**
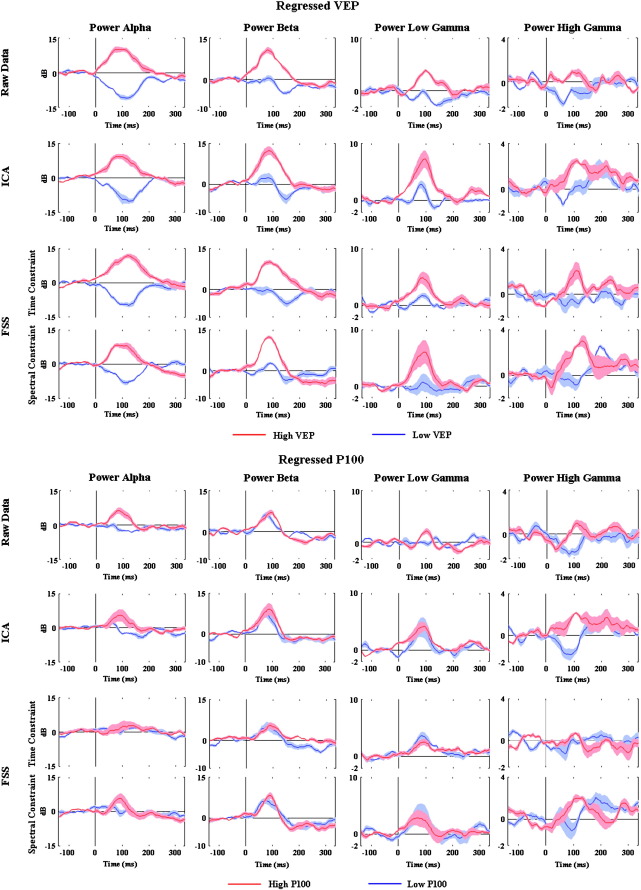
Frequency comparison between trials with high and low VEP/P100 areas. Upper half—the first (solid blue line) and fourth (solid red line) quartiles of the trials ordered according the VEP area. The envelope indicates plus and minus one standard deviation around the mean. Lower half**—**as described above but for the P100 area.

To emphasize these effects, Fig. 7 shows the upper and lower quartiles of the data presented in columns 2 and 3 of Fig. 6 for the regressed data (i.e., the 25% of trials with the highest VEP/P100 area, and the 25% of trials with the lowest VEP/P100 area). In Fig. 7, when the evoked component was regressed out, higher power in alpha, beta and low/high gamma band can be seen for the fourth quartile with respect to the first quartile when we ordered by the VEP (Fig. 7 upper half), with respect to the P100 (Fig. 7 lower half). Fig. 7 also demonstrates more clearly that these effects were more evident when ordering by the VEP area than by the P100 area, suggesting a link specifically between VEP area and spectral power in the alpha, beta and gamma bands. Note that as the data in Fig. 7 were baseline corrected in the interval − 100 ms to 0 ms, the positive and negative deviations are variations about the mean. In particular for the alpha band, there is a prolonged desynchronisation that is evident throughout the stimulus period (see Figs. 3 and 4) which then becomes the baseline for the single trial analysis of Fig. 7. Superimposed on these sustained effects are transient peaks associated with the checkerboard reversal. These reversal-related alterations in the frequency content of the EEG can most clearly be seen in Fig. 4, and are present for all of the frequency bands examined. Fig. 7 therefore suggests that trials with a low VEP amplitude have proportionately more alpha desynchronisation than trials with a high VEP.

## Discussion

This study examined the ability of blind and semi-blind source separation techniques to extract sources specifically related to the Visual Evoked Potential (VEP/P100) and Gamma Band Activity (GBA) elicited by a reversing checkerboard. The relationship between the low frequency VEP and the high frequency (~ 30–90 Hz) GBA, both of which can be generated by simple visual stimuli such as checkerboards and gratings, remains unclear. GBA is receiving an increasing amount of attention because of its purported links with many different brain processes (Gray et al., 1989; Fries et al., 2001; Pesaran et al., 2002; Womelsdorf et al. 2006; see Tallon-Baudry, 2003, Ward, 2003 and Fries et al., 2007 for reviews). Of particular relevance to this study, high frequency EEG activity has been linked with early visual processing (Tzelepi et al., 2000; Sannita et al., 2001; Hadjipapas et al., 2007; Perfetti et al., 2007), a domain studied more commonly using the VEP (Di Russo et al., 2001, 2005). Despite the considerable amount of work characterising the behaviour of both the VEP and the GBA to manipulations of stimulus properties, very little attention has been paid to the relationship between the two. For example, it is not known whether they are different manifestations of the activity of a single neuronal pool, or whether they represent the stimulus responses of independent but spatially co-localised populations. A better understanding of this relationship is important to provide a more holistic approach to the analysis of EEG and MEG data, rather than a strict parcellation of different parts of the signal. It is also crucial for a proper interpretation of the results of combined EEG-fMRI recordings, which to date have focussed almost exclusively on low frequency EEG and evoked potentials, despite the fact that it has been suggested that the primary relationship between EEG and the Blood Oxygenation Level Dependent (BOLD) effect measured with fMRI is in the gamma band.

GBA has a very low signal-to-noise ratio (SNR), meaning that methods to improve its detectability and allow a more accurate characterisation of its properties are desirable. Three methods were examined in this study: Independent Component Analysis (ICA) and Functional Source Separation (FSS) with temporal and spectral constraints. ICA is a blind source separation technique that extracts sources based purely on statistical grounds, whilst FSS can be seen as an extension to ICA which incorporates some additional information to bias the decomposition algorithm towards solutions that satisfy physiological assumptions. In this study, a temporal constraint maximised the activity around the P100 of the VEP, and a spectral constraint maximised the difference in the GBA between the rest and the task periods. The purpose of applying these different analysis methods was threefold. Firstly, to determine which technique was able to provide the best characterisation of the GBA. Secondly, to provide a degree of validation to the comparison of the GBA and the VEP. Each of the techniques extracts a different part of the raw signal which is dependent on the assumptions underlying the decomposition. In particular, the ICA and the $EEG_{\mathrm{FS\gamma}}$ sources were explicitly intended to identify activity in the gamma band, whilst the $EEG_{\mathrm{FS}P100}$ source employed a temporal constraint designed to maximise the VEP. If similar conclusions regarding the relationship between the VEP and GBA are drawn from examination of these different sources then some confidence can be gained that they represent a realistic interpretation of the underlying data. Finally, the use of FSS with multiple constraints allows the relationship between the constraints to be studied directly. In this case, the temporal constraint centred on the P100 of the VEP selectively identified the most probable generator of that peak. The question can then be asked as to whether there is any evidence that this source also generates GBA. Conversely, a completely orthogonal spectral constraint centred on the gamma band was used to select the generator of the GBA, and the low frequency behaviour of that source examined. The ability of FSS to selectively identify specific sources has previously been demonstrated (Barbati et al., 2006, 2008; Porcaro et al., 2008, 2009, 2010; Tecchio et al., 2007, 2008; Betti et al., 2009) and, especially given the very different constraints employed in the current study, there is no reason to suspect that the two extracted sources should artefactually be related. That is, only if there is a genuine relationship between the VEP and the GBA will the activity of the two sources be similar. The results from this study provide clear evidence that the neuronal pools generating the VEP and GBA have a close spatial and statistical relationship. Localisation of the blind (ICA) and the semi blind method (FSS with both temporal and spectral constraint) was very consistent, with equivalent current dipoles (ECD) demonstrating very precise spatial co-localisation (Fig. 1 third column). This level of overlap supports the idea that the VEP and GBA are generated by spatially concordant neuronal populations. In particular, the comparison of the waveforms, localisation and time-frequency dynamics for the FSS with temporal and spatial constraint revealed a high degree of symmetry: maximising the signal extraction for the low frequency VEP lead to a source containing strong GBA, whilst maximising for the GBA resulted in a source with a clear VEP.

In order to characterise the GBA in more detail, and specifically to determine whether it was phase locked to stimulus presentation (i.e. evoked or induced), the event-related spectral perturbation (ERSP) was calculated in two ways (Fig. 3). Firstly, ERSP was calculated for each single trial, after which the spectra were averaged, and secondly the data were averaged in the time domain before performing spectral analysis. The second procedure maintains only the evoked (stimulus phase-locked) part of the GBR, whilst the first procedure will include both evoked and induced contributions. The results indicate a clear induced GBA for the semi blind methods with the spectral constraint, which is less apparent but also present in the other two data sets (Fig. 3 third column), in addition to an evoked component. This behaviour is consistent with previous work, particularly with grating stimuli (Hall et al., 2005; Muthukumaraswamy et al., 2010; Hadjipapas et al., 2007; Barbati et al., 2008).

A concern when using source separation techniques is whether in selecting a few ICs or only one FS some of the signal of interest has been lost. This was addressed in Fig. 2, which summarises the discrepancy (i.e. the difference between the raw data and the FSS or ICA data). According to this metric, the residual signal not extracted by FSS or ICA is comparable to the level of the baseline, indicating that most of the signal of interest was kept. In the same figure, it is demonstrated that the discrepancy results from $EEG_{IC_{k}}$ and $EEG_{\mathrm{FS}P100}$ are comparable, whilst the discrepancy of the $EEG_{\mathrm{FS\gamma}}$ is lower than the other two methods. It is of note that although the constraint used in the $EEG_{\mathrm{FS\gamma}}$ decomposition was specifically targeted towards the gamma activity, it appears from the discrepancy results that all of the peaks of the VEP were also extracted into the $EEG_{\mathrm{FS\gamma}}$ method. This is further evidence that the VEP and the GBA are generated by similar neuronal pools.

These analyses point to a robust relationship between the VEP and the GBA on average. To investigate this further, and to examine other frequency bands to determine whether the VEP is solely related to the gamma band, the relationship was examined on a single trial basis. Trials were ordered based on properties of the VEP (normalised area of the VEP or normalised area of the P100 peak), and the frequency content examined. If there is a link between the VEP and the frequency content of the EEG, there should be a difference between the frequency content of the highest and lowest quartiles of the ordered data. Figs. 5, 6 and 7 show that this is indeed the case. Specifically, there is a clear increase in alpha, beta and low/high gamma power for the top quartile of trials when ordered by VEP/P100 area. However, when the average evoked response was regressed out of the data, this effect was maintained only for the data ordered according to VEP area and not for data ordered by P100 amplitude. In the latter case, the effect of ordering was drastically reduced especially for the beta and gamma bands, whilst having much less of an effect on the alpha band. Taken as a whole, these results suggest that there is a robust relationship between the induced beta and low/high gamma activity and the VEP, indicating that this effect is not a result of contamination of the time-frequency analysis by fast components of the evoked potential.

**Figure S1:**
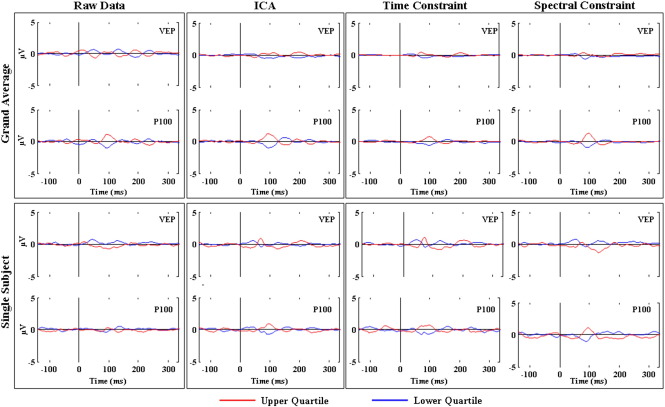
Residual activity of the regressed data. Grand Average (Upper Half) and a representative subject (Lower half) of the residual activity after regressing out the evoked response for both, VEP and P100, ordering methods.

Although it is very difficult to guarantee that all of the evoked component has been removed by the regression process, and as stated previously it explicitly assumes the standard model of evoked potential generation which is a matter of considerable debate (Min et al., 2007; Hanslmayr et al., 2007, Becker et al., 2008, Mazaheri and Jensen, 2006), several lines of evidence suggest that the majority of the evoked component has been taken out of the data (see Figure S1). The evoked model of VEP generation has been shown to be appropriate for early components of the VEP (< 175 ms, Becker et al. 2008), and the fact that the regressed data contains very little VEP in the single trial plots of Figs. 5 and 6 would support this. As is evident from Figs. 5 and 6, there is very little latency or shape variability in these early peaks of the VEP, which should at least give the best chance of removing the evoked component successfully (see also Figure S1).

This result may also have implications within the context of EEG-fMRI recordings, and in particular regarding a previous observation that the area of the VEP was correlated with the area of the BOLD response, but that the amplitudes were not related (Porcaro et al., 2010). Given the wealth of evidence that has been accumulated suggesting that the BOLD response is most strongly correlated with gamma band EEG (Logothetis et al., 2001, Niessing et al., 2005, Muthukumaraswamy and Singh, 2008, Zaehle et al., 2009), it is perhaps surprising that EEG-fMRI studies of evoked potentials have had such success. One potential explanation suggested by the results presented in this study is that the evoked potential acts as a surrogate marker for induced gamma activity. At the single trial level, variability in the area of the evoked potential was correlated with variability in the GBA in the time window 0–200 ms (Figs. 5–7), perhaps representing a mechanism whereby evoked potentials could link with BOLD. The relationship between scalp EEG and BOLD remains to be clarified, and this putative intervening link remains speculative and open to further investigation, and is no doubt complicated by the presence of a more prolonged gamma band response throughout the stimulation time period of five seconds (see Fig. 4, FSS Spectral Constraint in the low gamma band). For example, it would be important to determine whether the observations that have been made in primary visual cortex extend to other cortical regions, although the ideal scenario of examining these issues with concurrent EEG-fMRI is complicated by the current difficulty in cleanly recording high frequency activity in the MRI scanner (Mullinger et al., 2008).

In this study, the improvement in data quality provided by ICA and FSS facilitated a more detailed investigation of the spatiotemporal properties of the pattern-reversal EEG response than is generally possible. GBA was not clearly seen in the Raw data (Fig. 3 first row), perhaps explaining why the gamma band response to checkerboard stimuli has not been widely investigated. This improvement in the extraction and characterisation of particular signal properties allowed the relationship between the GBA and the low-frequency VEP to be investigated, the results of which suggested common behaviour and potentially common generators of these two well-studied phenomena. Future work could investigate the effects of stimulus properties on this link, for example static grating stimuli which are more effective at generating GBA (Hadjipapas et al., 2007) as well as higher order cognitive processes such as attention (Womelsdorf and Fries, 2007). More generally, the methodology proposed here suggests a way in which the links between the two main methods of analysing EEG and MEG data could be explicitly investigated to provide a much more complete view of the brain's response to external stimuli.

The following are the supplementary materials related to this article.

## Figures and Tables

**Table 1:** Explained variance.

	Num. ICs	Exp.Var. (%)
S1	30	98.75
S2	36	98.22
S3	39	95.87
S4	20	98.71
Mean	31	97.9
SD	8.4	1.4

For each subject, the number of estimated ICs (Num. ICs) and the Explained Variance (Exp.Var.) are shown after the dimensionality reduction procedure (see text). In the last two rows the mean and standard deviation (SD) across subjects are shown.

**Table 2:** Selected electrodes, Latency and Discrepancy Index.

	**EEG**_*IC*_*k*__			**EEG**_*FSP*100_			**EEG**_*FSγ*_		
Channel	Latency	DI	Channel	Latency	DI	Channel	Latency	DI
S1	Ch12-POO1	95	3.8	Ch12-POO1	94	6.7	Ch12-POO1	95	3.5
S2	Ch15-PPO2h	78	1.5	Ch 25-PO6h	82	2.2	Ch 25-PO6h	83	1.5
S3	CH22-POO2	99	4.6	Ch 20-OI2h	97	4.0	Ch25-PO6h	99	1.4
S4	Ch15-PPO2h	84	2.5	Ch15-PPO2h	83	1.5	Ch15-PPO2h	83	2.3
G.A.	Ch12-POO1	95	2.0	Ch12-POO1	96	4.3	CH22-POO2	95	1.6

For each subject and for each methodology, the selected channel, latency of the P100 and the Discrepancy Index (DI) are shown. The last row shows the same data for the Grand Average (G.A.).

**Table 3:** Dipole localisation.

	x	y	z	r.v.
**EEG**_*IC*_*k*__	− 4 [5.17]	− 62 [2.16]	25 [6.40]	1.58 [4.81]
**EEG**_*FSP*100_	1[1.60]	− 65 [8.03]	30 [6.55]	1.64 [0.89]
**EEG**_*FSγ*_	3 [8.56]	− 73 [0.63]	27 [3.49]	3.75 [2.66]

For each method (**EEG**_*IC*_*k*__,**EEG**_*FSP*100_and **EEG**_*FSγ*_), the mean position (x, y, and z) [standard error] and residual variances (r.v.) of the corresponding ECD are reported.
